# Parental food provisioning behaviours and perceptions in relation to environmentally sustainable diets for young children

**DOI:** 10.1093/heapro/daag025

**Published:** 2026-03-05

**Authors:** Nuvini Samarathunga, Alison C Spence, Carley A Grimes, Catherine G Russell, Kathleen E Lacy

**Affiliations:** Institute for Physical Activity and Nutrition (IPAN), School of Exercise and Nutrition Sciences (SENS), Deakin University, 75 Pigdons Road, Waurn Ponds, Victoria 3216, Australia; Institute for Physical Activity and Nutrition (IPAN), School of Exercise and Nutrition Sciences (SENS), Deakin University, 75 Pigdons Road, Waurn Ponds, Victoria 3216, Australia; Institute for Physical Activity and Nutrition (IPAN), School of Exercise and Nutrition Sciences (SENS), Deakin University, 75 Pigdons Road, Waurn Ponds, Victoria 3216, Australia; Institute for Physical Activity and Nutrition (IPAN), School of Exercise and Nutrition Sciences (SENS), Deakin University, 75 Pigdons Road, Waurn Ponds, Victoria 3216, Australia; Institute for Physical Activity and Nutrition (IPAN), School of Exercise and Nutrition Sciences (SENS), Deakin University, 75 Pigdons Road, Waurn Ponds, Victoria 3216, Australia

**Keywords:** sustainable diet, children, environmental sustainability, parents, Australia

## Abstract

Promoting environmentally sustainable diets for children is crucial for human and planetary health now and for future generations. Much research has examined parents’ roles in shaping children's dietary behaviours from a nutrition viewpoint, but there is little evidence regarding whether and how parents consider environmental sustainability in feeding their children. This study primarily aimed to understand parental perceptions, current practices, motivation, and self-efficacy regarding environmentally sustainable diets for children. A cross-sectional online survey was conducted among Australian parents of children aged 2–8 years (*n* = 316), recruited via social media and a university website. The survey included closed-ended and open-ended questions, with quantitative data analysed using descriptive statistics and chi-square tests, while thematic analysis was applied to open-ended responses. Australian parents’ perceptions aligned with many aspects of environmentally sustainable diets, with the majority highlighting that providing local and seasonal foods aligns with such a diet. Most parents were already engaging in multiple sustainable food provisioning and purchasing behaviours (60%–90%), such as providing fruit and vegetables in season (95%) and avoiding buying food without knowing how it will be used (90%). Although fewer parents engaged in behaviours such as providing fruits and/or vegetables from a home or community garden (60%) and actively searching for products in degradable, compostable, or recyclable packaging (65%), there was high motivation and self-efficacy to adopt them. Understanding perceived enablers and barriers to parents providing children with sustainable diets is an important next step in designing interventions to support parents in providing healthy and sustainable diets for their children.

Contribution to Health Promotion

- Parents shared how they think about and provide environmentally sustainable diets for young children (2–8 years), offering insight into a life stage where parental influence is critical in shaping dietary behaviours.
- Most parents were engaging in environmentally sustainable food provisioning and purchasing behaviours, while many of those who were not reported high motivation and self-efficacy to adopt behaviours.
- This study provides comprehensive insight into which environmentally sustainable food behaviours parents are engaging in, and where gaps exist between motivation and actual practices, which can inform the development of targeted and effective health promotion interventions to support families with young children.

## Introduction

In recent years, there has been a focus on moving the population towards an environmentally sustainable diet, driven by concerns that the current global food system has significant adverse effects on both human health and the physical environment (Food and Agriculture Organization of the United Nations 2017, Fanzo and Davis 2019). The growing world population is increasing the demand for food, thereby putting pressure on natural resources and contributing to environmental degradation through deforestation, water pollution, greenhouse gas emissions, and biodiversity loss, ultimately impacting climate change (Pimentel 1991, Freudenberg and Nestle 2020). Furthermore, in many countries, current diets are unhealthy and contributing to the development of non-communicable diseases such as type 2 diabetes and coronary heart disease, which adversely affect life expectancy (Tilman and Clark 2014). The importance of combining considerations of sustainability and nutritious diets has been a focal point of the United Nations, which introduced the 17 Sustainable Development Goals in 2015 (United Nations 2015). Concurrently, the EAT-Lancet Commission focused on the necessity of shifting global dietary patterns towards environmentally sustainable diets and established global dietary guidelines to achieve this goal (Willett *et al*. 2019).

Environmentally sustainable diets, as defined by the Food and Agriculture Organization of the United Nations (FAO), are those that have a low environmental impact and ensure food and nutrition security for present and future generations, supported by food systems that integrate the interconnected domains of health, environment, economics, and society (Food and Agriculture Organization of the United Nations 2012, Drewnowski *et al*. 2020). An environmentally sustainable diet encompasses both dietary components and food provisioning behaviours (Friel *et al*. 2014, Pearson *et al*. 2014, Willett *et al*. 2019, World Health Organization 2019). Dietary components (i.e. foods consumed), as defined by the FAO and the EAT-Lancet Commission, prioritize plant-based foods, including wholegrain products, fruits, vegetables, legumes, nuts, and seeds, while limiting highly processed foods and including moderate amounts of animal-based and dairy foods (Friel *et al*. 2014, Pearson *et al*. 2014, Willett *et al*. 2019, World Health Organization 2019). Environmentally sustainable food provisioning behaviours include reducing food waste and minimizing energy use in food purchasing, storage, and cooking, as well as food purchasing behaviours, such as purchasing seasonal and local produce and products with minimal packaging (Friel *et al*. 2014, Pearson *et al*. 2014, Willett *et al*. 2019, World Health Organization 2019).

The World Health Organization has identified the negative impact of current food systems on diets and health of infants and children, who are in a critical phase for growth and development that lays the foundation for lifelong human health and planetary health (Birch and Fisher 1998, Anzman-Frasca *et al*. 2018, United Nations International Children's Emergency Fund 2020, Halicka *et al*. 2021). This crucial life stage is an opportunity to develop healthy and environmentally sustainable eating habits that influence dietary intake and health throughout the life course and support the health of the planet (Scaglioni *et al*. 2018). During early childhood, parents have a major influence on children’s diets and the development of food choices through the purchasing and provision of food, and as role models in shaping children’s dietary behaviour (Savage *et al*. 2007, Mahmood *et al*. 2021). Therefore, understanding parental perceptions and behaviours related to the provision of environmentally sustainable diets for their children is essential, given parents’ central role in food decision-making during early childhood and their influence on shaping dietary behaviours that support the transition to a more sustainable food system (Halicka *et al*. 2021, Vos *et al*. 2022).

Motivation (i.e. internal processes that energize and direct behaviour, including habitual responses, emotions, and conscious decision-making) and self-efficacy (i.e. a person's belief in their own ability to successfully perform a specific behaviour) are known to influence behaviours, as shown by the Capability, Opportunity, Motivation-Behaviour (COM-B) model and Social Cognitive Theory (Bandura 1997, Michie *et al*. 2011). These constructs have been identified as important in explaining health behaviours and have also been shown to explain environmentally sustainable dietary behaviours among adults but have not been explored in the context of parental provision of sustainable diets for children (Graça *et al*. 2023, Arrazat *et al*. 2024, Rickerby and Green 2024). These frameworks help in understanding these influences, in the context of parental provision of sustainable diets for children, which is important to inform future interventions to promote behaviour change.

To date, limited studies have focused on parental perceptions of environmentally sustainable diets and food provisioning behaviours for their children. A small study conducted in Belgium involving 78 parents of children aged 6–12 years identified that parents perceived healthy food choices to be more important than sustainable ones and lacked knowledge about environmentally sustainable diets (Vos *et al*. 2022). Conversely, a larger study conducted in Poland involving parents (*n* = 1050) of children aged 6–8 years identified that 73% of the sample rated sustainability factors as important when purchasing food for their children, such as purchasing locally produced and seasonal foods and using reusable packaging (Halicka *et al*. 2021). These two studies provide valuable insights into parental attitudes and behaviours; however, both were conducted among parents of school-aged children and focused only on selected aspects of environmentally sustainable diets, such as sustainability considerations when purchasing food or discussing food and environment topics with children, without examining parents’ broader perceptions of environmentally sustainable diets or their everyday food-provisioning behaviours (Halicka *et al*. 2021, Vos *et al*. 2022). Together, these findings suggest potential international differences, but no previous Australian study has examined parents’ perceptions and behaviours related to environmentally sustainable diets for children, particularly among parents of younger children, who may be more influenced than older children by parental choices (Haerens *et al*. 2009, Peters *et al*. 2013, Halicka *et al*. 2021, Vos *et al*. 2022).

Sociodemographic characteristics, such as age, education level, and gender play a significant role in shaping individuals’ engagement with environmentally sustainable diets (Ammann *et al*. 2023). Studies from the UK and Belgium have shown that sociodemographic characteristics play a significant role in shaping individuals’ engagement with environmentally sustainable diets (Culliford and Bradbury 2020, Vos *et al*. 2022). Knowing whether such demographic associations also apply in Australia is important to inform future interventions aimed at supporting environmentally sustainable food provisioning among parents (Vos *et al*. 2022, Forray *et al*. 2023). The aims of the current study were to understand parental perceptions of environmentally sustainable diets, as well as current practices, motivation and self-efficacy related to environmentally sustainable food provisioning behaviours. A secondary aim was to understand how sociodemographic factors relate to parents’ environmentally sustainable food provisioning behaviours.

## Methods

### Participants and study design

A cross-sectional survey with mixed quantitative and open-ended items was used, allowing parents to describe their experiences in their own words. This approach, used previously in health research (Goode *et al*. 2023, González-Sánchez *et al*. 2024) enables the inclusion of a large number of participants, and is particularly useful for investigating topics among time-poor groups such as parents with young children. An online survey was conducted via Qualtrics (Provo, UT, USA) and targeted parents of children aged 2–8 years who were living in Australia and responsible for providing food for their child. Participants were recruited between August and November 2024 through paid and non-paid advertisements on Facebook, Instagram, X, and a university website with the aim of recruiting a diverse sample of parents of children aged 2–8 years. Ethics approval was obtained by the Deakin University Human Ethics Advisory Group (Project No: HEAG-H 109_2024). Following completion of the survey, respondents were given the opportunity to enter a prize draw to win one of five $50 supermarket e-vouchers, which were awarded through random selection.

### Online survey

Those who clicked on the survey link embedded in the survey advertisements were directed to the screening page, where they completed five eligibility questions (i.e. parent of child aged 2–8 years, living in Australia, competent in basic English and entirely or partially responsible for providing food for their child). Eligible participants were provided with a downloadable version of the Plain Language Statement and Consent Form. Those who consented were then directed to the rest of the online survey. Parents who reported having more than one child aged 2–8 years were asked to focus on the youngest child when answering questions.

The survey was created to explore Australian parents’ perceptions, current practices, motivation, self-efficacy, and perceived barriers and enablers to providing environmentally sustainable diets for children, with the intention to identify modifiable factors and inform future intervention approaches. The survey was designed to draw upon existing relevant measures but apply these to a sample of parents. The survey consisted of 48 closed-ended and 11 open-ended questions covering (i) demographic characteristics, (ii) environmentally sustainable diets, and (iii) environmentally sustainable food provisioning (i.e. providing and purchasing) behaviours, using questions modelled on several previous studies (Tobler *et al*. 2011, Pearson *et al*. 2014, Mann *et al*. 2018, Żakowska-Biemans *et al*. 2019, Brennan *et al*. 2020, Kansal *et al*. 2022). The dietary and food-related behaviours included in the survey were informed by definitions of an environmentally sustainable healthy diet proposed by the EAT-Lancet Commission report (2019) and FAO as well as definitions from two studies conducted in Australia (Friel *et al*. 2014, Pearson *et al*. 2014, Willett *et al*. 2019, World Health Organization 2019).

This study includes data obtained from three open-ended and sixteen closed-ended questions related to parents’ perceptions of environmentally sustainable diets as well as current practices, motivation and self-efficacy. The remaining survey questions related to perceived barriers and enablers to providing a sustainable diet are reported elsewhere.

Three open-ended questions were used to assess parents’ perceptions about environmentally sustainable diets for children: ‘What is the first thing that comes into your mind when you hear the term an ‘environmentally sustainable diet’ for children?’, ‘In your opinion, what types of foods would your child’s diet include to align with an “environmentally sustainable diet”?’ and ‘In your opinion, what types of foods would your child’s diet not include to align with an “environmentally sustainable diet”? These questions were adapted from a previous study conducted in Australia to gain an in-depth understanding of adults’ (not necessarily parents) perspectives on environmentally sustainable eating patterns (Mann *et al*. 2018). However, the open-ended questions were modified to investigate parental perceptions to fit the context of this study (Mann *et al*. 2018). These questions were placed in the survey before all other sustainability questions to collect parents’ initial top-of-mind thoughts about the topic, in their own words.

Sixteen closed-ended questions analysed in this study assessed food provisioning and purchasing behaviours related to: limiting highly processed foods (2 items), providing seasonal and local produce (5 items), providing products with minimal packaging (4 items) and minimizing food waste (5 items) (Pearson *et al*. 2014, Mann *et al*. 2018, Żakowska-Biemans *et al*. 2019, Brennan *et al*. 2020, Kansal *et al*. 2022) (Supplementary file S1). Five questions related to limiting highly processed foods and providing seasonal and local produce for their children were adapted from a study that used both exploratory qualitative and quantitative methods to develop an instrument to measure self-reported consumer sustainable healthy eating behaviours (Żakowska-Biemans *et al*. 2019). Additional questions regarding providing seasonal and local produce were taken from two Australian studies that identified priority areas for changing behaviour related environmentally sustainable and healthy food choices and explored adults’ perspectives on sustainable eating patterns (Pearson *et al*. 2014, Mann *et al*. 2018). Five questions related to food waste were adapted from a study of parents’ perceptions of children's influence on household food waste, which collected qualitative data from 15 focus groups of Australian households (Kansal *et al*. 2022). Furthermore, four questions related to minimizing packaging were adapted from a survey conducted in Australia to understand consumer perceptions and understanding of packaging (Brennan *et al*. 2020).

The 16 closed-ended questions used a 6-point scale with response options adapted from a previous study designed to assess consumer intentions to adopt environmentally sustainable diets (Tobler *et al*. 2011). The adapted responses in this study included: ‘I am already doing this most of the time’ (often behaviour), ‘I am already doing this sometimes’ (sometimes behaviour), ‘I am already doing this occasionally’ (occasionally behaviour), ‘I am not doing this; I would like to and believe I could make this change’ (no behaviour plus high motivation and high self-efficacy), ‘I am not doing this; I would like to but believe it is too hard or I don’t know how’ (no behaviour plus high motivation and low self-efficacy), and ‘I am not doing this; I don’t want to do this or I don’t know how’ (no behaviour plus no motivation and no self-efficacy) (Tobler *et al*. 2011).

Nine closed-ended questions assessed demographic characteristics of parents and their children. The demographic questions collected information on age, country of birth, and gender for both children and parents, number of children in the household, number of children aged 2–8 years living in the household and education level of the parent completing the survey. Additionally, one question assessed where parents or other household members typically shop for food for their child. The survey structure and content were reviewed by members of the research team, including experienced researchers in nutrition and public health, to ensure clarity, relevance, and content validity. Pilot testing with 10 adults of varying demographic backgrounds (age, gender, and education level) resulted in minor revisions to improve readability and reduce the time required to complete the survey to approximately 20 minutes.

### Statistical analysis

Data were downloaded from Qualtrics into STATA 17.0 and cleaned. Duplicate IP addresses, duplicate email addresses, survey completion times of less than 5 minutes, suspicious or implausible responses to closed-ended questions (e.g. reporting five or more children aged 2–8 years in a household), temporal events (e.g. defined as responses submitted between 1:00 a.m. and 4:00 a.m. or surveys that were started at the same time and date), and reCAPTCHA scores were used to identify potentially unreliable responses. Data obtained from the closed-ended questions in the survey were analysed using STATA 17.0. Descriptive statistics were used to summarize participants’ characteristics and their response to each of the closed-ended questions in the survey.

For analysis, the six-point response scale for items about current practices, motivation and self-efficacy to adopt environmentally sustainable food provisioning behaviours was grouped into two categories. Responses indicating regular engagement, including ‘I am already doing this most of the time’, ‘I am already doing this sometimes,’ and ‘I am already doing this occasionally’, were classified as ‘Doing this behaviour’. All other responses were classified as ‘Not doing the behaviour’. The association between behaviour engagement and sociodemographic factors was examined using the Chi-square test, with statistical significance defined as *P* < 0.05.

Given that most parents provided brief, single-sentence responses to open-ended questions, conventional content analysis was used to systematically describe and quantify patterns within the data (Hsieh and Shannon 2005). Data were read and re-read for familiarization and an initial round of coding was conducted by the first author using NVivo to identify initial themes (Lumivero 2023). A 20% subset of responses from three open-ended questions was independently coded by a co-author. Coders discussed any additional themes and reached an agreement on the final codebook. To ensure intercoder reliability, coded data were compared, and any discrepancies were resolved through discussion until agreement was achieved (Campbell *et al*. 2013, O’Connor and Joffe 2020). The remaining 80% of responses were then coded by NS. After completing the coding process, all authors reviewed the identified themes to ensure they accurately reflected the data and were appropriately labelled. Further, quantitative content analysis was undertaken to count the frequency and percentage of identified themes (Bengtsson 2016). Responses that mentioned multiple relevant themes were coded under all applicable themes.

## Results

### Characteristics of participants

A total of 316 participants were included in the final analysis. Of the 462 responses to the survey, 121 responses were excluded because respondents consented but did not answer any study-related questions, 17 incomplete responses (<50% of the survey questions related to this study were completed) were excluded, and eight potentially unreliable responses (e.g. reCAPTCHA score <0.5, duplicate IP addresses and email addresses, and completion time <5 minutes) were excluded. The majority of participants were women (94%) and aged 35–44 years (65%). Most were born in Australia (72%), and a high proportion (84%) had a university degree. The most common number of children in the household was two (46%), and 66% of respondents had one child aged 2–8 years. Among children, 50% were male, and 94% were born in Australia. Most parents (88%) reported being primarily responsible for grocery shopping, while 12% shared this responsibility. Participants’ characteristics are presented in Table 1.

**Table 1:** Demographic characteristics of participants (*n* = 316).

Characteristics	*N* (%)
**Parent characteristics**	
Age	
18–24 years	2 (0.6%)
25–34 years	91 (28.8%)
35–44 years	206 (65.2%)
45–54 years	17 (5.4%)
55+ years	0
Gender	
Woman	298 (94.3%)
Man	14 (4.4%)
Gender diverse	3 (1.0%)
Prefer not to answer	1 (0.3%)
Country of birth	
Australia	228 (72.2%)
Main English-speaking countries (Canada, Ireland, NZ, South Africa, UK, US)	27 (8.5%)
Other	60 (19.0%)
Prefer not to answer	1 (0.3%)
Education level	
Postgraduate qualifications	6 (1.9%)
University degree	267 (84.5%)
Trade, Apprenticeship, Diploma, Certificate	29 (9.2%)
Year 12	9 (2.8%)
Year 11 or below	4 (1.3%)
Prefer not to answer	1 (0.3%)
Number of children in household	
1	113 (35.8%)
2	144 (45.6%)
3 or more	59 (18.6%)
Number of children in 2–8 age group in household	
1	207 (65.5%)
2	99 (31.3%)
3	8 (2.5%)
4	2 (0.6%)
Responsibility for grocery shopping for the child	
Mainly responsible for food grocery shopping	278 (88.0%)
Share the responsibility for food grocery shopping	38 (12.0%)
**Child characteristics (2–8 years)**	
Age	
2–3 years	145 (45.9%)
4–8 years	171 (54.1%)
Gender	
Male	158 (50.0%)
Female	153 (48.4%)
Prefer not to say	5 (1.6%)
Country of birth	
Australia	297 (94.0%)
Main English-speaking countries (Canada, Ireland, NZ, South Africa, UK, US)	3 (1.0%)
Other	15 (4.7%)
Prefer not to say	1 (0.3%)

Supermarkets were the primary food shopping location, with most parents shopping there at least weekly. Other fresh produce retailers (e.g. fruit and vegetable shop and bakery) were used by approximately a quarter of parents at least weekly, and about a third shopped at a butcher/fish shop at least fortnightly. In contrast, few parents regularly shopped at wholesalers (e.g. Costco), bulk ‘natural foods’ shops, farmers’ markets or direct from farmers, or used home delivered produce or meal boxes. The frequency of shopping for children from different types of retailers is presented in Supplementary Table S1.

### Thematic analysis

**Figure 1:**
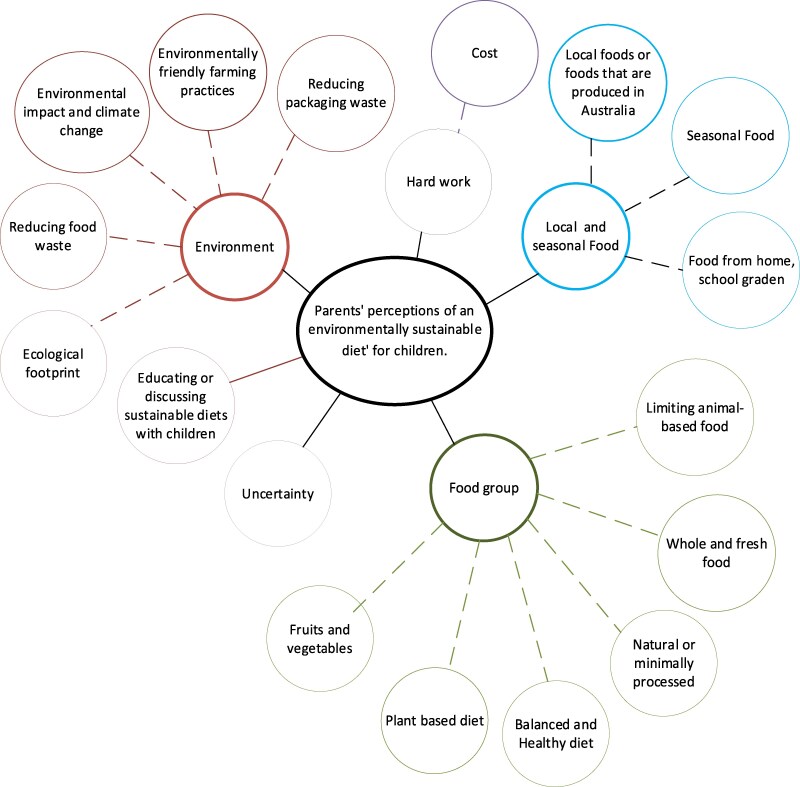
Parents’ perceptions of environmentally sustainable diets for children in Australia. Each colour represents a different theme, and line thickness of each bubble reflects the frequency of each theme. Solid lines represent key themes, while dashed lines represent sub-themes. Themes mentioned by <1% of participants were not included in the figure.

Most parents responded to each open-ended item with one short statement (e.g. 5 words), though some listed multiple sustainability considerations. Six primary themes emerged from the participants’ perspective of environmentally sustainable diets and the foods they believed would either align or not align with such diets for their children. These included environment, food groups, local and seasonal food, educating or discussing sustainable diets with children, hard work, and uncertainty regarding environmentally sustainable diets for children. These themes and subthemes are visually represented in Fig. 1 and further exemplified through parental quotes in Supplementary Tables S3–S5. Themes mentioned by less than 1% of participants are not presented.

#### Theme 1: Environment

The most identified theme was environment, with 61% (*n* = 193) of parents aware that food choices contribute to environmental impact and climate change. Some participants described environmentally sustainable foods as those with a lower ecological footprint, referring to aspects such as carbon emissions or environmentally friendly farming practices. Additionally, some parents emphasized that a sustainable diet involves reducing food waste and minimizing packaging waste to reduce environmental impact. For example, one participant stated:

‘Choosing foods that have a low carbon footprint’

#### Theme 2: Food groups

More than half of parents (59%, *n* = 186) described an environmentally sustainable diet for children based on its dietary components. The majority emphasized a balanced and healthy diet that includes whole and fresh foods, plant-based options, and minimally processed foods. Parents particularly highlighted food groups that align with an environmentally sustainable diet for children, such as wholegrains, fruits, vegetables, and legumes, while highly processed and large quantities of animal-based products were reported as not aligning with an environmentally sustainable diet for children. Participants who discussed animal products in the context of an environmentally sustainable diet for children indicated either choosing more sustainable options (e.g. wild meat, kangaroo, fish) or locally sourced and free-range products or considering them within the overall diet. For example, one participant stated:

‘When I hear “environmentally sustainable diet” for children, I think of a diet that emphasizes foods with a lower environmental impact. This would typically include more plant-based foods, such as fruits, vegetables, legumes, and whole grains, and reduced consumption of animal products, particularly those with high resource use and greenhouse gas emissions’.

#### Theme 3: Local and seasonal food

Fifty percent of parents (51%, *n* = 161) described their perception of an environmentally sustainable diet for children as one that includes regional or locally produced foods, or those grown in Australia. Furthermore, some parents emphasized that foods sourced from home, community, or school gardens contribute to sustainability by reducing environmental impact. Others highlighted the importance of providing seasonal foods for their children, recognizing that consuming in-season produce reduces food miles. For example, one participant stated:

‘Choose locally sourced, seasonal produce to reduce transportation emissions’

#### Theme 4: Educating or discussing sustainable diets with children

A few parents (3%, *n* = 9) recognized that educating or discussing sustainable diets with children is an essential component of an environmentally sustainable diet, emphasizing the importance of teaching children about the environmental impact of their food choices. For example, one participant stated:

‘Educating children on the environmental impact of their food choices’.

#### Theme 5: Hard work

A few parents (3%, *n* = 10) indicated that an environmentally sustainable diet is challenging, describing it as ‘hard work’ and not always feasible. Cost was another major concern, with parents expressing concerns about the affordability of sustainable food choices. For example, one participant stated:

‘How can this be achieved in a cost sustainable way?’

#### Theme 6: Uncertainty

A small proportion (2%, *n* = 6) of parents expressed uncertainty about the concept of an environmentally sustainable diet, indicating a lack of awareness or understanding of its key components, principles, and implications for food choices. For example, one participant stated: ‘I have no idea what that is’.

### Current practices, motivation and self-efficacy for environmentally sustainable food provisioning and purchasing behaviours

The most frequently reported environmentally sustainable food provisioning behaviour was providing fruit and/or vegetables in season, with 69% of parents reporting doing this most of the time. Other frequently reported behaviours included providing foods produced in Australia (55%) and saving a child’s leftovers for consumption at another meal (50%). In contrast, the least frequently reported behaviours were providing fruits and/or vegetables from a home or community garden (21% most of the time) and providing regional or local foods (28% most of the time). For food provisioning behaviours, about one in five parents (19%) reported high motivation and high self-efficacy for providing fruits and/or vegetables from a home or community garden.

The most commonly reported environmentally sustainable food purchasing behaviour was avoiding buying food without knowing what it will be used for, with 66% of parents reporting doing this most of the time. Other frequently reported behaviours included buying fresh food loose rather than packaged (54%) and avoiding impulse purchases influenced by marketing or shelf placement (47%). In contrast, the least frequently reported behaviours were actively searching for products in degradable, compostable, or recyclable packaging (23% most of the time) and buying food products not transported over long distances (23% most of the time). For food purchasing behaviours, about one in five parents (21%) reported high motivation and high self-efficacy to search for products in degradable, compostable, or recyclable packaging.

**Figure 2:**
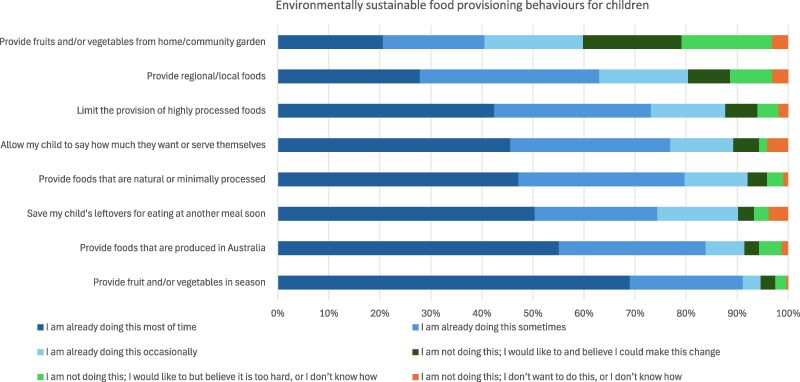
Proportion of parents’ engaging in environmentally sustainable food provisioning behaviours or reporting motivation and self-efficacy for these behaviours (*n* = 316).

**Figure 3:**
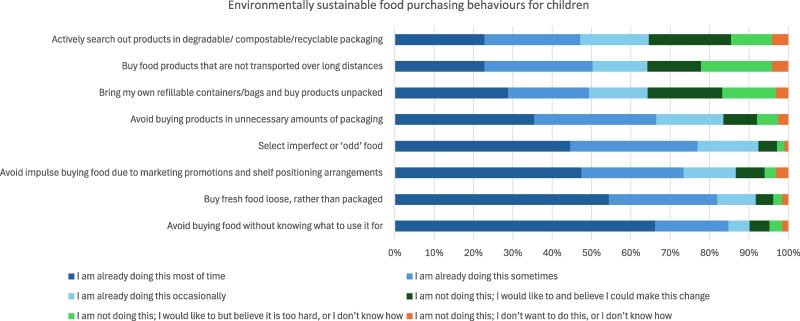
Proportion of parents’ engaging in environmentally sustainable food purchasing behaviours or reporting motivation and self-efficacy for these behaviours (*n* = 316).

Across all food provisioning and purchasing behaviours, between 50% and 90% of parents were already engaging in these behaviours. Although fewer parents engaged in some behaviours, there was high motivation and self-efficacy to adopt them, while only a small proportion (around 5%) reported not engaging in the behaviours and having low motivation and low self-efficacy (Figs 2 and 3).

### Parents’ environmentally sustainable food provisioning behaviours and associations with sociodemographic characteristics

Few behaviours were associated with sociodemographic factors. Parents aged 35–54 years were more likely than those aged 18–34 years to avoid buying food without knowing what to use it for (*P* = 0.015), bring refillable containers when shopping and buy unpackaged products (*P* = 0.006), allow children to serve themselves (*P* = 0.047), and provide seasonal fruits and vegetables (*P* = 0.029). Additionally, child age was negatively associated with providing foods produced in Australia (*P* = 0.010). Parental education level and country of birth, and number of children in the household, were not significantly associated with any of the assessed behaviours. Associations of parents’ environmentally sustainable food provisioning behaviours with sociodemographic characteristics are presented in Supplementary Table S2.

## Discussion

This study provides the first comprehensive investigation into Australian parental perceptions, current practices, motivation and self-efficacy for environmentally sustainable food provisioning behaviours for their children aged 2–8 years. Parents’ perceptions aligned with accepted scientific concepts of an environmentally sustainable diet for children, describing it as a balanced and healthy diet that also has a low environmental impact. Parents particularly emphasized the provision of plant-based foods such as whole grains, fruits, vegetables and legumes, while limiting highly processed, and animal-based foods. The majority of parents (>50%) highlighted that providing local and seasonal food aligned with an environmentally sustainable diet for children. Over 80% of parents were either already engaging in or reported high motivation and self-efficacy to adopt environmentally sustainable food provisioning and purchasing behaviours, whereas approximately 5% reported low motivation and low self-efficacy for these behaviours. Some differences in these perceptions, motivations and behaviours were observed according to demographic characteristics.

When examining parents’ perceptions of a sustainable diet for children, their responses were generally consistent with the scientific understanding of an environmentally sustainable diet, describing it as balanced, healthy, and low in environmental impact (Vos *et al*. 2022). There are no known studies specifically exploring parents’ perceptions of environmentally sustainable diets for children; however, a study in Belgium exploring the determinants of both healthy and sustainable food choices among parents of children 6–12 years (*n* = 78) identified a general lack of knowledge about environmentally sustainable diets (Vos *et al*. 2022). In the Australian context, no studies have specifically explored parents’ perceptions. In 2018, a study involving Australian adults aged 19–69 years identified a limited understanding of environmentally sustainable diets (Mann *et al*. 2018). Yet, when a similar study was conducted in 2022, the majority of young Australians (18–25 years) were aware of some aspects of sustainable and healthy diets, including the need to limit meat consumption, increase plant-based food intake, minimize food waste and packaging, and reduce food miles (Ronto *et al*. 2022). In the present study, overall, the combined responses of the parents were generally consistent with the principles of environmentally sustainable diets, though some uncertainty remained. The responses offered may indicate topics of most interest, relevance or awareness among parents such as providing local and seasonal products, reducing food waste, and minimizing packaging, and could inform framing and focus areas for future interventions. Although awareness of sustainable diets is important, its translation into consistent behaviours is not assured and may be influenced by factors such as self-efficacy and motivation (Bandura 1997, Ajzen 1991).

A notable proportion of participants emphasized the importance of including fruit and vegetable consumption, along with the inclusion of grains, whole grains, legumes, and nuts, while limiting ultra-processed and discretionary food, which aligns well with the dietary components suggested by the EAT-Lancet Commission and Australian Dietary Guidelines (National Health and Medical Research Council 2013, Willett *et al*. 2019). However, despite this alignment in parental perceptions, an analysed dietary data from the 2011–2012 National Nutrition and Physical Activity Survey for Australian children in the same age group (2–8 years), identified that the daily mean consumption of whole grains, vegetables, legumes, and nuts was substantially lower than the recommended intakes according to the Australian Dietary Guidelines and the Planetary Health Diet targets, while the intake of discretionary foods and beverages was higher than recommended (Samarathunga *et al*. 2024). This discrepancy suggests a gap between parents’ perspective of an environmentally sustainable diet for children and actual dietary practices. Understanding the underlying factors such as the enablers and barriers to adopting an environmentally sustainable diet for children is essential to addressing this gap.

Nearly all participants who discussed meat in the context of an environmentally sustainable diet for children indicated either limiting meat consumption, choosing more sustainable options, or considering meat within the overall diet reflecting a consistent awareness of the environmental impact of meat. Furthermore, parents highlighted strategies to incorporate lower-impact animal products, such as poultry, eggs, and sustainable fish, alongside increasing plant-based protein sources (legumes, nuts, and seeds). Specific to the Australian context, some parents also mentioned including kangaroo, which is classified as red meat in the Australian Dietary Guidelines and is considered more environmentally sustainable given its lower greenhouse gas emissions compared to other red meats (National Health and Medical Research Council 2013, Ratnasiri and Bandara 2017). This is consistent with findings from Australian young adults, who were familiar with limiting meat consumption in alignment with an environmentally sustainable diet (Ronto *et al*. 2022). Further research is needed to investigate the role of meat in environmentally sustainable diets, particularly for vulnerable groups such as children, as reducing meat intake without appropriate planning may compromise children’s intake of essential nutrients such as iron, zinc, and vitamin B12, given their high requirements for growth and development (Leroy and Barnard 2020, Neufingerl and Eilander 2023). Furthermore, understanding parents’ perspectives on the enablers and barriers to providing meat within an environmentally sustainable diet, is an important next step in identifying key behavioural influences.

A moderate proportion of parents reported mostly engaging in, or expressed high motivation and self-efficacy to adopt, behaviours related to minimizing food waste. Specific behaviours parents reported to minimize food waste included saving their child's leftovers for eating at another meal soon (42%, most of the time), avoiding impulse buying food (48%, most of the time), avoiding buying food without knowing what to use it for (66%, most of the time), and selecting imperfect or ‘odd’ food (45%, most of the time). Despite parents engaging in these positive behaviours related to food waste, few parents identified food waste as a key component of an environmentally sustainable diet in their open-ended responses. These findings align with a cross-sectional study conducted in Australia, which identified that having children influences parents to engage in impulse buying, and make ‘just in case’ food purchases, behaviours that contribute to increased food waste (Kansal *et al*. 2022). The results highlight that while many parents recognize the importance of minimizing food waste, practical challenges are encountered and there is relatively low recognition of food waste reduction as an environmental sustainability practice. These findings underscore the importance of increasing awareness among parents, particularly as reducing food waste may be a more achievable and less complex behaviour compared to limiting consumption of animal products, with fewer potentially adverse nutritional consequences, especially for nutritionally vulnerable groups such as young children.

Furthermore, the findings indicate that parents aged 35–54 years were more engaged in behaviours related to reducing food waste and packaging compared with younger parents. Similarly, a UK study identified that consumers over 35 years of age were more concerned about food waste compared with younger participants (Culliford and Bradbury 2020). Research on food waste highlights that younger consumers are more likely to generate food waste due to concerns over freshness, improper storage, excessive purchasing, and frequent shopping (Bravi *et al*. 2019, Ghinea and Ghiuta 2018). In contrast, older consumers may have greater skills and knowledge in meal planning and utilizing leftover food, which helps reduce waste (Thyberg and Tonjes 2016, Bravi *et al*. 2019). It is important to raise awareness across all age groups, particularly among families with children, given younger children significantly influence food waste generation in households (Karunasena *et al*. 2021, Kansal *et al*. 2022).

Similar to reducing food waste, a high proportion (83%–96%) of parents reported mostly engaging in, or expressed high motivation and self-efficacy to adopt, behaviours to reduce packaging waste, although only a moderate proportion (22% −54%) reported practising these behaviours most of the time. However, certain behaviours related to reducing packaging waste, such as bringing their own refillable containers/bags and buying products unpacked (28%) as well as actively searching out products in degradable/compostable/recyclable packaging (∼23%), were less commonly practiced. These results align with a study in Poland, where most parents considered sustainability when buying food, including the use of reusable packaging (Halicka *et al*. 2021). Additionally, a study conducted with Australian young adults found that the majority of participants were aware of the need to reduce food packaging (Ronto *et al*. 2022). Most parents in the present study reported shopping primarily at supermarkets, where options to reduce packaging may be limited in comparison to bulk food stores or farmers’ markets, where they shopped infrequently. Additionally, the packaging of healthy and environmentally sustainable foods, such as fruits and vegetables, in excessive packaging may create confusion among parents. This may be particularly challenging for parents, as some of these packaged products are specifically marketed towards children (e.g. ‘child-sized’ apples in plastic bags or ‘child-sized’ bananas bundled with bands). While packaging plays a crucial role in reducing food waste by preserving and protecting food throughout transport and storage (Williams and Wikström 2011), there is a need to understand strategies to minimize excessive packaging through sustainable packaging materials and to understand barriers limiting parents’ adoption of packaging-reduction behaviours, despite high motivation and self-efficacy.

Fifty percent of parents highlighted that providing local and seasonal food aligns with an environmentally sustainable diet for children and around 60% currently engage in behaviours related to providing local and seasonal food, such as purchasing food products that are not transported over long distances and providing fruits and/or vegetables from a home/community garden. A similar study in Poland revealed that most parents consider environmental sustainability when purchasing food, such as purchasing locally produced and seasonal foods (Halicka *et al*. 2021). Similarly, research among young Australian adults has shown that being local and in season are important considerations when making food choices (Ronto *et al*. 2022). These findings suggest that the perceived importance of local and seasonal food may offer a useful entry point for engaging parents in broader conversations about environmentally sustainable diets.

Furthermore, the findings indicate that parents of younger children were less engaged in providing foods produced in Australia than parents of older children, although there is limited literature available to directly compare results based on child age. Notably, many parents referred to ‘Australian’ produce, which may reflect a general preference for nationally sourced foods rather than a more specific understanding of local or regional sourcing. In geographically large countries such as Australia, foods labelled as Australian can still travel long distances, meaning that truly local foods produced within the same region often have lower transport-related emissions (Coley *et al*. 2009). Clarifying the distinction between local and national food origins in food labelling and retail environments may be an important area for future attention, particularly to support parents in making informed decisions about truly local food options that strengthen local economies, enhance social equity in line with food systems that integrate the interconnected domains of health, environment, economics, and society and contribute to lower environmental impacts (Feenstra 1997, Food and Agriculture Organization of the United Nations 2012, Drewnowski *et al*. 2020).

Australian parents’ perceptions align with many aspects of environmentally sustainable diets as defined by the FAO (Food and Agriculture Organization of the United Nations 2012, World Health Organization 2019). However, other key aspects, such as ensuring adequate but not excessive nutrient intake and including safe and clean drinking water as the fluid of choice were less recognized. This may show geographical differences (i.e. safe, clean drinking water is freely available in Australia) and highlights the need for further exploration of what comprehensively constitutes a sustainable diet for children and the barriers to effectively translating these principles into everyday meals.

A key strength of this study was its exploration of a previously underexamined topic on parental perceptions of environmentally sustainable diets for children, combined with a methodological approach that integrates researcher-driven questioning with responses in participants’ own words, thereby providing quantitative data alongside initial parental perceptions. The collection of participants’ perspectives on environmentally sustainable diets for children, and on which foods align or do not align with such diets, captured at the start of the survey and in their own words, offers a unique opportunity to gather broader insights from a large sample of parents. Furthermore, this study explored both dietary and food provisioning behaviours using a broader approach that has been examined in a limited number of studies, contributing to a more holistic understanding of sustainable food provision among parents.

An online survey open to parents from across Australia was used with the intention to recruit a large, varied sample of parents. While the sample size was relatively large, there were limited data in certain demographic subgroups, with the majority of participants being aged 34–54 years, predominantly female, and highly educated. Approximately 50% of women aged 25–44 in the general Australian population hold a bachelor’s degree or above, and similar patterns have been reported in nutrition research, (Burnett *et al*. 2023, Australian Bureau of Statistics 2025) suggesting that the predominantly highly educated sample in the present study may not fully reflect the broader population of Australian parents. This sample may adopt different strategies compared to the general population, as it is possible that highly educated parents may have greater awareness (Ammann *et al*. 2023). Future research should target recruitment to parents with lower levels of education to further investigate these findings. Additionally, the study was based on self-reported data, which presents the potential for social desirability bias, as participants’ responses may not fully reflect their actual environmentally sustainable food provisioning behaviours for children. Additionally, this study is limited in that it captured only brief open-ended survey responses from parents regarding their perspectives on environmentally sustainable diets, including which foods they perceived as aligning or not aligning with such diets for children. This approach enabled the collection of parents’ own words across a large sample, although it often constrained the depth of insight into their perceptions. Future research incorporating interviews or focus groups could provide richer and more detailed accounts.

## Conclusions

The results indicate that parents’ perceptions of an environmentally sustainable diet for children generally align with the scientific understanding of an environmentally sustainable diet. High proportions of parents were already engaging in behaviours such as providing fruit and/or vegetables in season and avoiding buying food without knowing how it will be used. About one in five parents reported high motivation and high self-efficacy for providing fruits and/or vegetables from a home or community garden (19%) and actively searching for products in degradable, compostable, or recyclable packaging (21%). Key aspects defined by the FAO, such as ensuring balanced nutrient intake were less recognized. These findings suggest that barriers may exist in effectively translating these principles into everyday meals for children.

A critical next step in promoting healthier and more environmentally sustainable diets for children involves further exploration of the enablers and barriers to adopting environmentally sustainable food provisioning behaviours through in-depth qualitative research. A deeper understanding these factors will provide valuable insights for developing targeted interventions that support parents in making informed, practical, and sustainable choices.

## Supplementary Material

daag025_Supplementary_Data

## Data Availability

The analysed data supporting the findings of this study are available in Supplementary files S1, S2, and S3.

## Data availability

The data underlying this article will be shared on reasonable request subject to ethical approval and in accordance with participant consent.
