# Supplementary material for: Parental food provisioning behaviours and perceptions in relation to environmentally sustainable diets for young children
Source: Health Promot Int. 2026 Mar 5;41(2):daag025. doi: 10.1093/heapro/daag025 (PMC13016778; doi:10.1093/heapro/daag025)
Supplement: daag025_Supplementary_Data [file daag025_supplementary_data.zip › Supplementary file 1 - Online Survey.docx]

Survey

Start the survey with a brief and clear introductory message.

“Thank you for your interest in our study. We are interested in finding out your thoughts about environmental sustainability when feeding children. Please complete a few questions below to check you can participate.”

The next page of the Qualtrics survey will contain the eligibility questions.

Q1. Are you currently living in Australia?

- Yes
- No

Q2. What is the postcode of your residential address?

________________________________________________________________

Q3. Are you the parent or guardian of at least one child aged 2-8-years (inclusive)?

- Yes
- No

Q4. Are you entirely or partially responsible for providing food for your child/children aged between 2 and 8 years?

- Yes, I’m entirely or partially responsible for providing food for my child/children aged 2-8 years.
- No, I’m not entirely or partially responsible for providing food for my child/children aged 2-8-years.

Q5. Are you competent in understanding and writing in basic English?

- Yes
- No

If a potential participant is not eligible (i.e. outside Australia, not competent in English and not responsible for providing food for their child/children aged 2-8-years), then they will receive the following thank-you message:

Thank you for your interest in participating in this study and for completing these questions. Unfortunately, your responses do not align with the inclusion criteria for the study at this time. We wish you all the best.

The next page of the Qualtrics survey will contain Plain Language Statement (PLS) and consent. This page will be titled “PLAIN LANGUAGE STATEMENT AND CONSENT FORM”, and at the end will ask participants to select “I understand the information supplied on the plain language statement. Completion and submission of this online survey signifies my consent to my data being used as outlined in the plain language statement or “No, I do not consent”. If participants provide consent to participate in the survey, the next page in Qualtrics will contain a hyperlink to a PDF version of the Plain Language Statement/consent form, which shows their consent status. The Withdrawal of Consent Form will be attached to the second part of the Plain Language Statement (PLS) for participants to keep if they have provided consent to participate in the survey.

**SECTION ONE – DEMOGRAPHIC INFORMATION**

In this section we are interested in finding out a little bit of background information about you and your child aged 2-8 years. This information will help us compare the answers of respondents from different backgrounds.

Q6. What is your age group?

- Under 18 years
- 18-24 years
- 25-34 years
- 35-44 years
- 45-54 years
- 55-65 years
- >65 years

If a participant is under 18 years, they will be directed to the end of the survey and will receive the thank-you message mentioned above.

Q7. What is your gender?

- Woman
- Man
- Gender Diverse
- Prefer to self describe __________________________________________________
- Prefer not to answer

Q8. In which country were you born?

- Australia
- Main English-speaking countries (Canada, Ireland, NZ, South Africa, UK, US)
- Other __________________________________________________
- Don’t know
- Prefer not to answer

Q9. What is the highest level of education you have completed?

- University Degree
- Trade, Apprenticeship, Diploma, Certificate
- Year 12
- Year 11 or below
- Other, please specify __________________________________________________
- Don’t know
- Prefer not to answer

Q10. How many children do you have living in your household?

- 1
- 2
- 3
- 4
- 5 or more

Q11. How many children aged 2-8 years (inclusive) live in your household?

- 1
- 2
- 3
- 4
- 5 or more

Q12. Thinking about the child/children aged 2–8 years in your care, what is the age of the youngest child in this age range? This is the child we want you to answer the remaining survey questions for.

- 2 years
- 3 years
- 4 years
- 5 years
- 6 years
- 7 years
- 8 years

Q13. What is the gender of this child? (e.g. your youngest child in the 2–8-years age group)

- Male
- Female
- Prefer not to say.

Q14. What is the country of birth of this child? (e.g. your youngest child in the 2–8-years age group)

- Australia
- Main English-speaking countries (Canada, Ireland, NZ, South Africa, UK, US)
- Other ________________________________________________
- Don’t know
- Prefer not to answer

Thank you for providing this background information on you and your child. **Please remember for the rest of the survey focus on your child aged 2-8 years. If you have more than one child aged 2-8 years, remember to focus on the youngest child identified above.**

Q15. Are you mainly responsible for food grocery shopping for your child?

- Yes
- No
- I share the responsibility

Q16.How often do you or other members of your household shop for foods for your child at each of these store types?

|  | Never | Less than monthly | Monthly | Fortnightly | Weekly | More than weekly |
| --- | --- | --- | --- | --- | --- | --- |
| Wholesale (e.g. Costco) |  |  |  |  |  |  |
| Supermarket (e.g. Coles, Woolworths, Aldi) including online order/delivery |  |  |  |  |  |  |
| Fruit and vegetable shop |  |  |  |  |  |  |
| Butcher/Fish shop |  |  |  |  |  |  |
| Bakery |  |  |  |  |  |  |
| Bulk “natural foods” shop |  |  |  |  |  |  |
| Direct from famers |  |  |  |  |  |  |
| Farmer’s market |  |  |  |  |  |  |
| Home-delivered meal boxes (e.g. EveryPlate, HelloFresh, Dinnerly) |  |  |  |  |  |  |
| Delivered produce boxes |  |  |  |  |  |  |
| Other (e.g. neighbours selling eggs) |  |  |  |  |  |  |

Q17. What is the first thing that comes into your mind when you hear the term an “environmentally sustainable diet” for children?

________________________________________________________________

________________________________________________________________

________________________________________________________________

________________________________________________________________

________________________________________________________________

Q18. In your opinion, what types of foods would your child’s diet include to align with an "environmentally sustainable diet”?

________________________________________________________________

________________________________________________________________

________________________________________________________________

________________________________________________________________

________________________________________________________________

Q19. In your opinion, what types of foods would your child’s diet **NOT** include to align with an "environmentally sustainable diet”?

________________________________________________________________

________________________________________________________________

________________________________________________________________

________________________________________________________________

________________________________________________________________

Q20. When **providing** food for your child, what are your current practices OR willingness to do the following in the next 6 months? **Remember focus on your youngest child in the 2–8-year-old age group.**

|  | I am already doing this most of time | I am already doing this sometimes | I am already doing this occasionally | I am not doing this; I would like to and believe I could make this change | I am not doing this; I would like to but believe it is too hard or I don’t know how | I am not doing this; I don’t want to do this or I don’t know how |
| --- | --- | --- | --- | --- | --- | --- |
| Limit the provision of highly processed foods (e.g. fast food and ready meals, salty snacks, crackers, sausages) |  |  |  |  |  |  |
| Provide foods that are natural or minimally processed |  |  |  |  |  |  |
| Provide regional/local foods (e.g. foods from within my state) |  |  |  |  |  |  |
| Provide foods that are produced in Australia |  |  |  |  |  |  |
| Provide fruit and/or vegetables in season |  |  |  |  |  |  |
| Provide fruits and/or vegetables from home/community garden |  |  |  |  |  |  |
| Save my child's leftovers for eating at another meal soon |  |  |  |  |  |  |
| Allow my child to say how much they want or serve themselves |  |  |  |  |  |  |

Q21. When **purchasing** food for your child, what are your current practices OR willingness to do the following in the next 6 months?

|  | I am already doing this most of time | I am already doing this sometimes | I am already doing this occasionally | I am not doing this; I would like to and believe I could make this change | I am not doing this; I would like to but believe it is too hard or I don’t know how | I am not doing this; I don’t want to do this or I don’t know how |
| --- | --- | --- | --- | --- | --- | --- |
| Buy food products that are not transported over long distances |  |  |  |  |  |  |
| Select imperfect or ‘odd’ food (e.g. smaller apples, bent carrots) |  |  |  |  |  |  |
| Bring my own refillable containers/bags and buy products unpacked |  |  |  |  |  |  |
| Avoid buying products in unnecessary amounts of packaging |  |  |  |  |  |  |
| Buy fresh food loose, rather than packaged |  |  |  |  |  |  |
| Actively search out products in degradable/ compostable/recyclable packaging |  |  |  |  |  |  |
| Avoid impulse buying food due to marketing promotions and shelf positioning arrangements |  |  |  |  |  |  |
| Avoid buying food without knowing what to use it for |  |  |  |  |  |  |

Q22. What do you think about when deciding which meat (e.g. red meat , poultry, fish) or meat alternatives (e.g. plant-based meat, tofu, eggs) to provide to your child?

________________________________________________________________

________________________________________________________________

________________________________________________________________

________________________________________________________________

________________________________________________________________

Q23. How often do you consider any environmental aspects when providing meat or meat alternatives to your child?

- Most of the time
- Sometimes
- Occasionally
- Rarely

Display Question 23:

If How often do you consider any environmental aspects when providing meat or meat alternatives to... = Most of the time

Or How often do you consider any environmental aspects when providing meat or meat alternatives to... = Sometimes

Or How often do you consider any environmental aspects when providing meat or meat alternatives to... = Occasionally

Q24. Please tell us about how you consider environmental aspects when providing meat or meat alternatives to your child.

________________________________________________________________

________________________________________________________________

________________________________________________________________

________________________________________________________________

________________________________________________________________

Display Question 24:

If How often do you consider any environmental aspects when providing meat or meat alternatives to... = Rarely

Q25. What makes it difficult to consider environmental aspects when providing meat or meat alternatives to your child?

________________________________________________________________

________________________________________________________________

________________________________________________________________

________________________________________________________________

________________________________________________________________

Q26. What do you think about when deciding which dairy foods (e .g. dairy milk, cream, cheese, yoghurt) or dairy alternatives (e.g. soy milk, rice milk) to provide to your child?

________________________________________________________________

________________________________________________________________

________________________________________________________________

________________________________________________________________

________________________________________________________________

Q27. How often do you consider any environmental aspects when providing dairy foods or dairy alternatives to your child?

- Most of the time
- Sometimes
- Occasionally
- Rarely

Display Question 27:

If How often do you consider any environmental aspects when providing dairy foods or dairy alternati... = Most of the time

Or How often do you consider any environmental aspects when providing dairy foods or dairy alternati... = Sometimes

Or How often do you consider any environmental aspects when providing dairy foods or dairy alternati... = Occasionally

Q28. Please tell us about how you consider environmental aspects when providing dairy foods or dairy alternatives to your child.

________________________________________________________________

________________________________________________________________

________________________________________________________________

________________________________________________________________

________________________________________________________________

Display Question 28:

If How often do you consider any environmental aspects when providing dairy foods or dairy alternati... = Rarely

Q29.What makes it difficult to consider environmental aspects when providing dairy foods or dairy alternatives to your child?

________________________________________________________________

________________________________________________________________

________________________________________________________________

________________________________________________________________

________________________________________________________________

Q30. How often do you think about energy use when food purchasing, storing or cooking food for your child? (e.g. do you walk to the shops instead of drive, or use cooking equipment that uses less power)

- Most of the time
- Sometimes
- Occasionally
- Rarely

Display Question 31:

If How often do you think about energy use when food purchasing, storing or cooking food for your ch... = Most of the time

Or How often do you think about energy use when food purchasing, storing or cooking food for your ch... = Sometimes

Or How often do you think about energy use when food purchasing, storing or cooking food for your ch... = Occasionally

Q31. Please tell us about how you consider energy use when purchasing, storing or cooking food for your child?

________________________________________________________________

________________________________________________________________

________________________________________________________________

________________________________________________________________

________________________________________________________________

Display Question 32:

If How often do you think about energy use when food purchasing, storing or cooking food for your ch... = Rarely

Q32. What makes it difficult to consider energy use when purchasing, storing or cooking food for your child?

________________________________________________________________

________________________________________________________________

________________________________________________________________

________________________________________________________________

________________________________________________________________

Q33. How often do you think about food waste when providing food for your child?

- Most of the time
- Sometimes
- Occasionally
- Rarely

Display Question 34:

If How often do you think about food waste when providing food for your child? = Most of the time

Or How often do you think about food waste when providing food for your child? = Sometimes

Or How often do you think about food waste when providing food for your child? = Occasionally

Q34. Please tell us about how you consider food waste when providing food for your child?

________________________________________________________________

________________________________________________________________

________________________________________________________________

________________________________________________________________

________________________________________________________________

Display Question 35:

If How often do you think about food waste when providing food for your child? = Rarely

Q35. What makes it difficult to consider food waste when providing food for your child?

________________________________________________________________

________________________________________________________________

________________________________________________________________

________________________________________________________________

________________________________________________________________

There’s no single or right definition of an environmentally sustainable diet for children. However, we could define an environmentally sustainable diet as one that:
  - contains mostly plant-based food (more whole grain products, fruit, vegetables, and legumes, nuts and seeds),
  - limits consumption of highly processed foods,
  - includes purchasing and consumption of seasonal and local produce,
  - includes purchasing products with minimal packaging,
  - reduces food waste, and
  - minimises energy use in food purchasing, storage and cooking.

Q36. Given this definition, what things might help you to feed your child an environmentally sustainable diet?

________________________________________________________________

________________________________________________________________

________________________________________________________________

________________________________________________________________

________________________________________________________________

Q37. Given this definition, what things might make it harder for you to feed your child an environmentally sustainable diet?

________________________________________________________________

________________________________________________________________

________________________________________________________________

________________________________________________________________

________________________________________________________________

Q38. If you would like to be eligible for prize notifications or receive a final summary of the study, please provide your email address.

- Email address __________________________________________________
- Skip

Q39. Would you like to:

- Be entered into the draw to receive a gift voucher.
- Receive a summary of the study findings.
