# Supplementary material for: Parental food provisioning behaviours and perceptions in relation to environmentally sustainable diets for young children
Source: Health Promot Int. 2026 Mar 5;41(2):daag025. doi: 10.1093/heapro/daag025 (PMC13016778; doi:10.1093/heapro/daag025)
Supplement: daag025_Supplementary_Data [file daag025_supplementary_data.zip › Supplementary_file_2-_Tables_of_Descriptive_Analysis_KL.docx]

Supplementary Table 1: The frequency of shopping for children from different types of retailers (n= 316)

|  | Never | Less than monthly | Monthly | Fortnightly | Weekly | More than weekly |
| --- | --- | --- | --- | --- | --- | --- |
| Wholesale (e.g. Costco) | 66.1% | 17.4% | 7.9% | 1.6% | 3.8% | 3.2% |
| Supermarket (e.g. Coles, Woolworths, Aldi) including online order/delivery | 0.9% | 2.2% | 1.6% | 7.0% | 50.9% | 37.3% |
| Fruit and vegetable shop | 16.1% | 22.1% | 13.0% | 15.2% | 23.4% | 10.1% |
| Butcher/Fish shop | 29.4% | 28.2% | 12.7% | 13.0% | 13.9% | 2.8% |
| Bakery | 19.6% | 25.9% | 17.7% | 10.4% | 19.0% | 7.3% |
| Bulk “natural foods” shop | 50.0% | 21.5% | 16.5% | 5.7% | 4.4% | 1.9% |
| Direct from famers | 58.5% | 23.7% | 5.4% | 6.3% | 4.1% | 1.9% |
| Farmer’s market | 41.5% | 35.1% | 11.1% | 7.0% | 4.1% | 1.3% |
| Home-delivered meal boxes (e.g. EveryPlate, HelloFresh, Dinnerly) | 78.5% | 13.6% | 2.2% | 2.2% | 1.9% | 1.6% |
| Delivered produce boxes | 74.7% | 12.0% | 3.2% | 5.1% | 4.1% | 0.9% |
| Other (e.g. neighbours selling eggs) | 57.0% | 23.7% | 8.2% | 4.4% | 5.1% | 1.6% |

Supplementary Table 2: Parents’ environmentally sustainable food provisioning behaviours and associations with sociodemographic characteristics

|  | Parents demographic characteristics | | | | | | | | | Household demographic characteristics | | | Child demographic characteristics | | | |
| --- | --- | --- | --- | --- | --- | --- | --- | --- | --- | --- | --- | --- | --- | --- | --- | --- |
|  | Age group (years) % (n=316) | | | Education level % (n=315) * | | | Country of birth% (n=316) | | | N children in house % (n=316) | | | Child age group (n=316) | | | |
|  | 18-34 | 35-54 | P-value | University degree | Non-University education | P-value | Born in Australia | Born outside Australia | P-value | one child | More than one child | *P*-value | 2-3 years | | 4-8 years | P-value |
| **Limit the provision of highly processed foods (e.g. fast food and ready meals, salty snacks, crackers, sausages)** | | | | | | | | | | | | | | | | |
| Doing this behaviour | 86% | 88% | 0.57 | 88% | 88% | 0.93 | 87% | 90% | 0.49 | 85% | 89% | 0.28 | 87% | 88% | | 0.70 |
| Not doing this behaviour | 14% | 12% |  | 12% | 12% |  | 13% | 10% |  | 15% | 11% |  | 13% | 11% | |  |
| **Provide foods that are natural or minimally processed** | | | | | | | | | | | | | | | | |
| Doing this behaviour | 89% | 93% | 0.3 | 92% | 90% | 0.88 | 90% | 97% | 0.06 | 89% | 94% | 0.18 | 94% | 90% | | 0.15 |
| Not doing this behaviour | 11% | 7% |  | 7% | 10% |  | 10% | 3% |  | 11% | 6% |  | 6% | 10% | |  |
| **Provide regional/local foods (e.g. foods from within my state)** | | | | | | | | | | | | | | | | |
| Doing this behaviour | 75% | 82% | 0.14 | 81% | 79% | 0.84 | 78% | 86% | 0.10 | 82% | 79% | 0.52 | 80% | 80% | | 0.89 |
| Not doing this behaviour | 25% | 18% |  | 19% | 21% |  | 22% | 14% |  | 18% | 20% |  | 20% | 20% | |  |
| **Provide foods that are produced in Australia** | | | | | | | | | | | | | | | | |
| Doing this behaviour | 88% | 93% | 0.18 | 91% | 93% | 0.89 | 90% | 95% | 0.11 | 90% | 92% | 0.58 | 96% | 88% | | **0.01** |
| Not doing this behaviour | 12% | 7% |  | 9% | 7% |  | 10% | 5% |  | 10% | 8% |  | 4% | 12% | |  |
| **Provide fruit and/or vegetables in season** | | | | | | | | | | | | | | | | |
| Doing this behaviour | 90% | 96% | **0.03** | 94% | 95% | 0.95 | 93% | 98% | 0.13 | 92% | 96% | 0.13 | 95% | 94% | | 0.69 |
| Not doing this behaviour | 10% | 4% |  | 6% | 5% |  | 7% | 2% |  | 8% | 4% |  | 5% | 6% | |  |
| **Provide fruits and/or vegetables from home/community garden** | | | | | | | | | | | | | | | | |
| Doing this behaviour | 59% | 60% | 0.87 | 60% | 57% | 0.67 | 61% | 57% | 0.50 | 64% | 58% | 0.29 | 59% | 61% | | 0.70 |
| Not doing this behaviour | 40% | 40% |  | 40% | 43% |  | 39% | 43% |  | 36% | 42% |  | 41% | 39% | |  |
| **Save my child's leftovers for eating at another meal soon** | | | | | | | | | | | | | | | | |
| Doing this behaviour | 90% | 90% | 0.96 | 91% | 83% | 0.26 | 90% | 91% | 0.79 | 89% | 91% | 0.72 | 92% | 88% | | 0.22 |
| Not doing this behaviour | 10% | 10% |  | 9% | 17% |  | 10% | 9% |  | 11% | 9% |  | 8% | 11% | |  |
| **Allow my child to say how much they want or serve themselves** | | | | | | | | | | | | | | | | |
| Doing this behaviour | 84% | 91% | **0.05** | 90% | 86% | 0.69 | 90% | 87% | 0.54 | 86% | 91% | 0.15 | 88% | 91% | | 0.38 |
| Not doing this behaviour | 16% | 9% |  | 10% | 14% |  | 10% | 13% |  | 14% | 9% |  | 12% | 9% | |  |
| **Buy food products that are not transported over long distances** | | | | | | | | | | | | | | | | |
| Doing this behaviour | 59% | 66% | 0.22 | 66% | 55% | 0.30 | 64% | 66% | 0.70 | 64% | 64% | 0.88 | 63% | 65% | | 0.61 |
| Not doing this behaviour | 41% | 34% |  | 34% | 45% |  | 36% | 34% |  | 36% | 36% |  | 37% | 35% | |  |
| **Select imperfect or ‘odd’ food (e.g. smaller apples, bent carrots)** | | | | | | | | | | | | | | | | |
| Doing this behaviour | 91% | 93% | 0.66 | 92% | 95% | 0.72 | 92% | 94% | 0.42 | 91% | 93% | 0.53 | 94% | 91% | | 0.39 |
| Not doing this behaviour | 9% | 7% |  | 8% | 5% |  | 8% | 6% |  | 9% | 7% |  | 6% | 9% | |  |
| **Bring my own refillable containers/bags and buy products unpacked** | | | | | | | | | | | | | | | | |
| Doing this behaviour | 53% | 69% | **0.01** | 64% | 62% | 0.72 | 64% | 66% | 0.70 | 60% | 66% | 0.27 | 61% | 67% | | 0.33 |
| Not doing this behaviour | 47% | 31% |  | 35% | 38% |  | 36% | 34% |  | 40% | 34% |  | 39% | 33% | |  |
| **Avoid buying products in unnecessary amounts of packaging** | | | | | | | | | | | | | | | | |
| Doing this behaviour | 80% | 85% | 0.37 | 83% | 83% | 0.90 | 84% | 83% | 0.86 | 80% | 85% | 0.28 | 85% | 82% | | 0.57 |
| Not doing this behaviour | 20% | 15% |  | 17% | 17% |  | 16% | 17% |  | 20% | 15% |  | 15% | 18% | |  |
| **Buy fresh food loose, rather than packaged** | | | | | | | | | | | | | | | | |
| Doing this behaviour | 90% | 92% | 0.54 | 93% | 88% | 0.62 | 92% | 92% | 0.91 | 88% | 94% | 0.11 | 94% | 89% | | 0.11 |
| Not doing this behaviour | 10% | 8% |  | 7% | 12% |  | 8% | 8% |  | 12% | 6% |  | 6% | 11% | |  |
| **Actively search out products in degradable/compostable/recyclable packaging** | | | | | | | | | | | | | | | | |
| Doing this behaviour | 61% | 66% | 0.43 | 66% | 55% | 0.28 | 64% | 65% | 0.96 | 66% | 64% | 0.61 | 61% | 67% | | 0.28 |
| Not doing this behaviour | 39% | 34% |  | 34% | 45% |  | 36% | 35% |  | 33% | 36% |  | 39% | 33% | |  |
| **Avoid impulse buying food due to marketing promotions and shelf positioning arrangements** | | | | | | | | | | | | | | | | |
| Doing this behaviour | 87% | 86% | 0.89 | 86% | 93% | 0.41 | 86% | 87% | 0.80 | 82% | 89% | 0.08 | 88% | 85% | | 0.45 |
| Not doing this behaviour | 13% | 14% |  | 14% | 7% |  | 14% | 12% |  | 18% | 11% |  | 12% | 15% | |  |
| **Avoid buying food without knowing what to use it for** | | | | | | | | | | | | | | | | |
| Doing this behaviour | 84% | 93% | **0.01** | 91% | 86% | 0.55 | 90% | 91% | 0.79 | 86% | 93% | 0.05 | 91% | 89% | | 0.64 |
| Not doing this behaviour | 16% | 7% |  | 9% | 14% |  | 10% | 9% |  | 14% | 7% |  | 9% | 11% | |  |

P-values less than 0.05 are considered statistically significant and are shown in bold.
