# Supplementary material for: Parental food provisioning behaviours and perceptions in relation to environmentally sustainable diets for young children
Source: Health Promot Int. 2026 Mar 5;41(2):daag025. doi: 10.1093/heapro/daag025 (PMC13016778; doi:10.1093/heapro/daag025)
Supplement: daag025_Supplementary_Data [file daag025_supplementary_data.zip › Supplementary_file_3_-Thematic_Analysis_KL.docx]

Supplementary Table 3: Parents' perceptions of an 'environmentally sustainable diet' for children.

| **Theme** | **Total ^a^ (N)** | **Example quotes** |
| --- | --- | --- |
| **Environment** | **193** |  |
| Environmentally friendly farming practices, with fish (n = 9) and organic food (n = 33) each explicitly mentioned | 68 | “Food that minimises harm to the environment through farming practices.”  “Sustainable seafood options”  “Organic food” |
| Environmental impact and climate change | 47 | “Food that doesn’t worsen climate change and affect sustainability negatively”  “Low climate impact” |
| Products with minimal packaging or reducing packaging waste/ environmentally friendly packaging | 42 | “Less packaged food”  “Environmentally friendly packaging” |
| Reducing food waste | 23 | “Cutting down food waste”  “Reducing the waste by meal planning” |
| Ecological footprint, including carbon emissions or carbon footprint. | 13 | “When I hear "environmentally sustainable diet" for children, I think of a diet that emphasizes foods with a lower environmental impact.”  “Low carbon foot print” |
| **Food group** | **186** |  |
| Whole and fresh food | 42 | “More whole foods”  “Using whole food” |
| Plant based diet | 36 | “Plant based foods”  “Plant based or plant focused diet,” |
| Foods that are natural or minimally processed | 32 | “no or minimally processed”  “less processing” |
| Limiting animal-based food | 29 | “limited animal-based foods”  “Less meat” |
| Balanced and Healthy diet | 25 | “Eating a balanced meal”  “Balanced nutrition: Ensure complete dietary needs.” |
| Fruits and vegetables | 22 | “More fruit and veg”  “Emphasize fruits and vegetables.” |
| **Local and seasonal food** | **161** |  |
| Regional/local foods or foods that are produced in Australia | 105 | “Shopping locally”  “Local, Short supply chain” |
| Food in season | 34 | “A diet that takes into consideration the seasonal availability of food”  ” Using produce that is in season” |
| Food from home/community garden/school grading | 22 | “Locally sourced or home grown as much as possible”  “My ideal is having our own homestead, which is our goal. I think growing our own food is the most.” |
| **Educating or discussing sustainable diets with children** | **9** | “Education: Teach kids about sustainability.”  “Educating children on the environmental impact of their food choices.” |
| **Hard work** | **10** |  |
| Hard work | 4 | “Hard work”  “Something to aim for, but not always the most practical option” |
| Cost | 6 | “How can this be achieved in a cost sustainable way?”  “Great if they’ll actually eat the food and it’s low cost” |
| **Uncertainty** | **6** | “Not sure”  “no idea” |

^a^ The total number of participants who reported each theme is presented. Themes mentioned by less than 1% of participants were not included in the table. If participants mentioned more than one theme, their responses were coded into all relevant themes where applicable.

Supplementary Table 4: Parents' perceptions on the types of foods comprising a child’s diet that align with an environmentally sustainable diet (n=316).

| **Theme** | **Subtheme** | **Align with an environmentally sustainable diet**  **N ^a^** | **Example quotes** |
| --- | --- | --- | --- |
| **Food group** |  |  |  |
| Fruits and vegetable |  | 184 | “Lots of fruit and veggies”  “vegetables and fruit” |
| Grains | Grain (Unspecified) | 26 | “grains”  “grains” |
|  | Whole Grain | 15 | “wholegrains”  “wholegrains” |
| Dairy and dairy products | Choosing more sustainable options and /or the context of whole foods | 19 | “dairy yogurt & cheese”  “fruit, veges, meat, dairy.” |
|  | Less or limited dairy products | 7 | “small amounts of local meat and dairy”  “Limited dairy but no eggs.” |
|  | Dairy alternatives | 8 | “Dairy Alternatives**: Options like almond, soy, or oat milk, especially if dairy production is a concern”  “Plant-based dairy” |
| Meat | Choosing more sustainable options and /or the context of whole foods | 52 | “Fruit and veggies eggs and meat”  “Meat vegies fruit” |
|  | Less or limited meat products | 18 | “Just a bit of red meat”  “Small amounts of local meat and dairy.” |
| Poultry | Choosing more sustainable options and /or the context of whole foods | 9 | “fruit and veggies, eggs and poultry”  “Fruit, vegetables, chicken” |
| Seafood | Choosing more sustainable options and /or the context of whole foods | 14 | “Fruit and vegetables, fish and meat”  “Vegetables and fruit, meat, fish” |
|  | Less or limited seafood | 5 | “ fish not too much.”  “occasional lean meats like poultry or fish.” |
| Legumes |  | 30 | “legumes”  “legumes, |
| Egg |  | 36 | “Egg”  “eggs” |
| Nuts and seed |  | 15 | “Nuts, seeds”  “Nuts, seeds” |
| Plant based diet |  | 17 | “Plant based foods”  “Primarily plant based” |
| Processed food | Minimally Processed or natural Foods | 29 | “Less processed foods”  “less processed food ” |
|  | Whole and Fresh food products | 23 | “Whole foods”  “Probably only whole foods” |
|  | Chemical-free and additive free food | 4 | “GMO, additive free”  “less sugar and preservatives” |
| Balanced and Healthy diet |  | 4 | “healthy and balanced”  “Food pyramid food” |
| **Environment** |  |  |  |
| Ethical Food Production |  | 8 | “Fair trade products to support ethical production and trade practices”  “ethical farmers.” |
| Friendly farming practices | Sustainable farming practices | 19 | “Foods from companies that prioritize sustainable agricultural practices and environmental conservation.”  “Sustainable farming practices from the producers such as biodynamic or permaculture farming” |
|  | Free range products | 9 | “free range animal products”  “Free range eggs” |
|  | Organic food | 30 | “organic produce”  “Organic.” |
| Minimise food waste |  | 6 | “produces less waste to make”  “We order are fruit and vegetable box from Farmers Pick that is aims to reduce food waste.” |
| Food packaging | Minimally Packaged and Unpackaged Foods | 26 | “foods without packaging.”  “ less packaging” |
| **Food origin** | Regional/local foods or foods that are produced in Australia | 114 | Locally sourced foods”  “Local produce” |
|  | Homegrown food | 32 | “Home grown vegetables and fruits.”  “We also grow some of our own herbs and vegetables.” |
|  | Home-Cooked and Homemade Foods | 7 | “Home cooked food.”  “homemade food.” |
| Seasonal food |  | 58 | “In season produce”  “in season” |

^a^ The total number of participants who reported each theme is presented. Themes mentioned by less than 1% of participants were not included in the table. If participants mentioned more than one theme, their responses were coded into all relevant themes where applicable.

Supplementary Table 5: Parents' perceptions on foods for a child’s diet that do not align with an environmentally sustainable diet (n=316)

| **Theme** | **Subtheme** | **Do not align with an environmentally sustainable diet**  **N^a^** | **Example quotes** |
| --- | --- | --- | --- |
| Food group | Dairy | 17 | “Dairy”  “Large amounts of meat and dairy” |
|  | Meat | 98 | “meat”  “Meat and animal products.” |
|  | Poultry | 5 | “caged bird produce”  “Not much white meat” |
|  | Seafood |  | “Most seafood.”  “No fish” |
|  | Egg | 5 | “eggs”  “eggs” |
|  | Processed food | 153 | “ultra processed foods”  “highly processed foods” |
|  | Discretionary food and Takeaway food | 91 | “Discretionary foods from the Australian dietary guidelines”  “Foods with excessive added sugars and unhealthy fats, which can contribute to health issues and environmental degradation through their production.” “Takeaway foods” |
|  | Palm oil | 8 | “Palm oil products”  “Anything containing palm oil” |
| Environment |  |  |  |
|  | Unsustainable Farming practices | 32 | “Unsustainably farmed.”  “Conventionally grown produce treated with harmful pesticides and herbicides.” |
|  | Genetically Modified Foods | 5 | “Genetically modified”  “Foods that contain genetically modified organisms (GMOs) if there are concerns about their environmental impact.” |
|  | Nonorganic food | 5 | “Non-organic produce”  “Non organic produce.” |
|  | Excessively Packaged Foods | 112 | “Foods with excessive packaging”  “Packaged foods” |
| Imported food |  | 92 | “Anything processed or imported from overseas”  “Anything imported” |
| Out of season |  | 20 | “Out of season”  “Out of season” |
| Uncertainty |  | 7 | “Not sure”  “Nothing” |

^a^ The total number of participants who reported each theme is presented. Themes mentioned by less than 1% of participants were not included in the table. If participants mentioned more than one theme, their responses were coded into all relevant themes where applicable.
